# Global Scale Variation in the Salinity Sensitivity of Riverine Macroinvertebrates: Eastern Australia, France, Israel and South Africa

**DOI:** 10.1371/journal.pone.0035224

**Published:** 2012-05-02

**Authors:** Ben J. Kefford, Graeme L. Hickey, Avital Gasith, Elad Ben-David, Jason E. Dunlop, Carolyn G. Palmer, Kaylene Allan, Satish C. Choy, Christophe Piscart

**Affiliations:** 1 Centre for Environmental Sustainability, School of the Environment, University of Technology Sydney, Broadway, New South Wales, Australia; 2 Northwest Institute for Bio-Health Informatics (NIBHI), University of Manchester, Manchester, United Kingdom; 3 Department of Zoology, Tel Aviv University, Tel Aviv, Israel; 4 Department of Environment and Resource Management, Brisbane, Queensland, Australia; 5 Institute of Water Research, Rhodes University, Grahamstown, South Africa; 6 Department of Zoology, University of Tasmania, Sandy Bay, Tasmania, Australia; 7 UMR CNRS 5023 Ecologie des Hydrosystèmes Naturels et Anthropisés, Université de Lyon 1, Villeurbanne, France; Federal University of Rio de Janeiro, Brazil

## Abstract

Salinity is a key abiotic property of inland waters; it has a major influence on biotic communities and is affected by many natural and anthropogenic processes. Salinity of inland waters tends to increase with aridity, and biota of inland waters may have evolved greater salt tolerance in more arid regions. Here we compare the sensitivity of stream macroinvertebrate species to salinity from a relatively wet region in France (Lorraine and Brittany) to that in three relatively arid regions eastern Australia (Victoria, Queensland and Tasmania), South Africa (south-east of the Eastern Cape Province) and Israel using the identical experimental method in all locations. The species whose salinity tolerance was tested, were somewhat more salt tolerant in eastern Australia and South Africa than France, with those in Israel being intermediate. However, by far the greatest source of variation in species sensitivity was between taxonomic groups (Order and Class) and not between the regions. We used a Bayesian statistical model to estimate the species sensitivity distributions (SSDs) for salinity in eastern Australia and France adjusting for the assemblages of species in these regions. The assemblage in France was slightly more salinity sensitive than that in eastern Australia. We therefore suggest that regional salinity sensitivity is therefore likely to depend most on the taxonomic composition of respective macroinvertebrate assemblages. On this basis it would be possible to screen rivers globally for risk from salinisation.

## Introduction

The salinity (the concentration of dissolved major inorganic ions) of inland waters has a large influence on the biotic communities [1], [2], [3] and naturally varies from 10′s of mg/L to a few 100’s of g/L [4]. Salinity of rivers is also affected by a number of anthropogenic activities including the clearing of native vegetation [5], [6], irrigation, weir pool depth [7], the salting of roads [8] and the discharges from mines [9], industry [3] and reverse osmosis effluent water re-cycling plants. Anthropogenic salinity rises have long been considered of globally importance [10]. The salinity of rivers in Queenland, Australia are affected by decadal scale temporal variation in climate [11] and salinity in many temperate streams is likely to increase under climate change [3], [12]. It is thus important to be able to assess the risk to freshwater species and their communities subject to increased salinity.

One method of assessing the risk of chemical contaminants to biota is to compile so called species sensitivity distributions (SSDs). SSDs are cumulative distribution functions of the (measured) sensitivity of a group of organisms to a chemical contaminant [13], in this case salinity. From a SSD it is possible to estimate the hazardous concentration (HC) for p% of species, often 5% and referred to as the HCp value. Conversely for a given salinity concentration, the potentially affected fraction (PAF) can be estimated. Such estimates from SSDs rely on a number of assumptions [14], [15]. For example, that the sample size of species sensitivity measurements is sufficiently large and adequately representative of the biological communities for which inferences are to be drawn.

There is a general lack of salinity sensitivity data in most parts of the world. Furthermore, the sensitivity data that do exist has been collected using a range of methods differing for example are the durations of exposure, life-stages exposed, salts used (sea water, NaCl, Na_2_SO_4_, etc.) and end-points measured [16]. Combining data collected using different methods into a SSD is problematic as species measured salinity sensitivity will depend on the method used. So as to increase the number of replicate species in SSDs, it would be of great practical value to use data from taxa living anywhere [17]. However, this requires the assumption that there is no regional variation in species salinity sensitivity.

Arguments have been advanced that certain continents or regions will tend to have freshwater biota which is more salt tolerant than elsewhere. In particularly, several studies have suggested that aquatic biota from Australia [18], [19] or, more generally, regions with (semi-)arid areas where saline waters are relatively common [20] should be more tolerant to salinity exposure than elsewhere. So, prior to conducting this study we expected salinity tolerances of regional freshwater assemblages to be correlated with the aridity of their region.

In this paper we compare the acute (72 h) lethal salinity sensitivity of stream macroinvertebrates to salinity, collected from freshwaters in eastern Australia, France, Israel and South Africa. In all localities species were tested using an identical method. Our chief aim was to determine if the salinity tolerances of the macroinvertebrate communities and taxonomic groups differ between the regions. Furthermore we consider whether it is reasonable to combine salinity sensitivity data from one part of the world with those from other parts and whether taxonomic composition of species included in the regional SSDs are representative of the taxonomic composition in respective regions.

## Methods

### The Data

The concentration of salt lethal to 50% of a population (LC_50_ values) over 72 h of salinity exposure was used here because they are relatively easy and quick to measure allowing for their determination across a large number of species [15]. Additionally 72 h LC_50_ values are correlated with the maximum salinity at which species have been recorded in nature [21], [22]. However, we stress that longer-term (chronic) mortality [23] and reduced growth and/or reproduction (sub-lethal effects) [24], [25], [26] occur at lower salinities than 72 h LC_50_ values. Additionally the eggs and hatchling of stream macroinvertebrates are more salt sensitive than older aquatic stages [27], [28]. Consequently, the 72 h LC_50_ values and parameters calculated from their distribution are not meant to be directly predictive of maximum salinity at which species of stream macroinvertebrate can maintain viable populations in nature.

Existing 72 h LC_50_ values of riverine macroinvertebrates from South Africa, in the south-east of the Eastern Cape Province [20] and eastern Australia from Tasmanian [29], south-west Victoria [16] and central Victoria [30] and four regions in Queensland [31] were compiled. These eastern Australian datasets were merged to form one dataset. The majority of species were unique to one of the eastern Australian regions but where a species occurred in multiple regions the criteria described in Hickey [32] was used to give one 72 h LC_50_ value per taxa. For example, a taxon with 72 h LC_50_ values of >30 mS/cm and 35–40 mS/cm in two regions would be given a value of 35–40 mS/cm here. New data (see Tables S1, S2, and S3) were collected from Lorraine and Brittany in France and in Israel from the following flowing waters: Avi’el, Divsha, Dora, Ga’ash, Hadera, HaShofet, Hermon, Tel-Aviv, Tut and Zuqim. Microinvertebrates (Branchiopoda, Ostracods, Copepods, Nematoda, Nematomorpha and Cnidaria) were excluded as their inclusion in the above datasets was variable and they are considerably more salt sensitive than macroinvertebrates [24]. The raw data and the species composition are available in the original publications for South Africa [20] and Eastern Australia [16], [29], [30], [31] and for the new French and Israeli data in Tables S1, S2, and S3.

In all regions a wide range of species was selected for testing in order to cover as many taxonomic groups that were present as practical. We attempted to select species for testing so that we had representative samples of species from each region. Artificial sea water was used as the salt source, with Ocean Nature (Aquasonic, Wauchope, NSW, Australia) used in Australia and South Africa and Instant Ocean (Red Sea Pharmaceuticals, Haifa, Israel) in France and Israel. An experiment was performed in Australia with two species with both of these artificial sea waters. It showed no significant difference in the 72 h LC_50_ values estimated for two species between Ocean Nature and Instant Ocean. The only difference in the method used was the temperature at which the experiments were conducted reflecting local climate. The South African and Victorian experiments were conducted at 20°C±2°C and 20°C±1°C, respectively. The Tasmanian experiments were conducted at 14°C (±1°C) and Queensland experiments at 25°C (±2°C). The French experiments were conducted at 18°C (±1°C) and the Israel experiments at 21°C (±1°C). The salinity sensitivities data in the various regions reflect tolerances under typical water temperatures at the sites and times when macroinvertebrates were collected.

### Data Analysis

#### Analysis of species tested from and between the regions

First we determined whether there are any differences in the distribution of species which happened to be tested in the four regions. Given the presence of right censored LC_50_ values (those>some value) SSDs were examined as Kaplan-Meier functions. Interval censored LC_50_ values (those estimated as between two values) were assumed to be the mid-point of the interval. The Log Rank (Mantel-Cox) test was used to compare for statistical significant differences between SSDs for each of the localities and between SSDs for the different taxa. This method was used as it involves the least assumptions. (We note that the major conclusions are unchanged with Breslow (Generalized Wilcoxon) and Tarone-Ware tests). Mean LC_50_ values (as estimated from the Kaplan-Meier functions) of different groups were considered to be statistically different when they had non-overlapping 95% confidence intervals.

The comparison of differences in LC_50_ values for particular taxa (mostly Classes or Orders) and regions was conducted using two methods. Firstly excluding right censored LC_50_ values, a two-factor ANOVA (taxa and regions) was used to compare 72 h LC_50_ values. However, there were two taxa (Crustaceans and Hydracarina) with less than two non-censored 72 h LC_50_ values per region. These taxa were excluded from the aforementioned two-factor ANOVA and analysed separately using single factor ANOVA (region). The 72 h LC_50_ values were square root transformed in order to better meet the assumptions of normality and homogeneity of variance required by ANOVA. These assumptions where checked by examining box-plots and q-q plots.

Secondly, using all data, for each taxa Kaplan-Meier functions were calculated from which mean 72 h LC_50_ values and 95% confidence intervals were compared. As before, Kaplan-Meier functions compared Log Rank (Mantel-Cox) test.

The mean and standard deviations of families and genera for each region of non-censored 72 h LC_50_ values were tabulated and compared non-statistically as there were too few species to allow for statistical tests.

#### Are the samples of species with LC50 values representative of their regions?

We estimated the proportion of stream macroinvertebrate species from each order in France and south-east Australia. (Israel and South Africa were not considered here as we were unable to make estimates in these countries.) This was done to determine if the species tested formed representative samples of the richness of orders in these two regions. As our interest was in considering spatial variation (on a global scale) in salinity sensitivity we did not consider any seasonal or other temporal variation in the presence of species within the regions. In France the number of flowing water species in each order was estimated from two sources: Limnofauna Europaea [33] and by counting the number of species listed in identification keys, literature and websites of naturalist organizations. The counts from both of these sources were similar (<1.3% discrepancy in the proportion of species in an order between the two sources), thus the means of both counts were used.

Similar checklists of species in Eastern Australia were not available; however the Victorian and South Australian Environmental Protection Authorities (EPA) have a database of species collected from 2966 samples from two habitats edge/pool and where present riffles across these states [1], [34]. We determined the number of species of each order that had been recorded in this database and used this as an estimate of the relative number of species in each order in Eastern Australia.

For the two regions (France and Eastern Australia), we determined if the percentage of species tested in a taxonomic order is similar to the percentage of species estimated to be in the relevant region. If sample of species with LC_50_ values was representative of their region then there should be a regression between these percentages close to the line y = x. That is if q% of the species from an order were tested, then ≈ q% of species in the region should be from this order.

#### Does the acute lethal salinity tolerance of stream macroinvertebrates differ between France and Eastern Australia?

**Figure 1:**
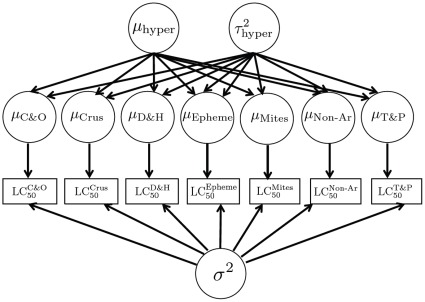
Bayesian network represented as a directed acyclic graph. Rectangular box nodes represent the 72-hr LC50 data for each group of species (in each box there will be at least one individual species measurements). Round nodes represent random variables: the subscript μ values denote the mean log10 72-hr LC50 for each group; σ^2^ is a measure of within-group interspecies variance; µ_hyper_ and τ^2^_hyper_ are the mean and variance of the hyper-population of group means. Directed arrows represent conditional probability statements. Prior distributions are placed over σ^2^, µ_hyper_ and τ^2^_hyper_.

We used a Bayesian statistical model, adapted from Hickey et al. [32], to determine if the SSD of stream macroinvertebrates assemblages differed between France and Eastern Australia. That is, do the actual distributions of sensitivity of the species in these regions differ, and not the species that happen to be tested in these regions? This model determines the statistical distribution of groups (or sub-sets) of the species and is represented in a directed acyclic graph in Figure 1. Each sub-set is weighted in terms of the species richness (or ecologically weighted) in France and Eastern Australia (as given above). The SSD that is calculated is then based on an ecological weighted sum of species-subset sensitivity distributions. The model assumes that on a log_10_ scale that each group of species has its own mean 72-h LC_50_ but that all groups have the same variance. The model is able to use both right and interval censored data without relying on the mid-point rule [32].

Here we used the following quasi-taxonomic groups: (1) Coleoptera and Odonata, (2) Crustaceans, (3) Diptera and Hemiptera, (4) Ephemeroptera, (5) Hydracarina, (6) non-arthropods, (7) Trichoptera and Plecoptera. These groups were chosen as analysis showed that most species within them had similar 72-h LC50 values and the variance in LC50 within these groups was broadly similar (see results). The only difference between the analysis used here and Hickey et al. [32] is that the latter used expert judgment of a group’s salinity sensitivity (on a scale of 0–10) to form a prior distribution of each group’s sensitivity, as adapted from Grist et al.[35]. Here we do not include expert judgments of salinity sensitivity, but rather assume that the quasi-taxonomic SSD means are exchangeable *a priori*. This is equivalent to the statement that we would not change our prior beliefs over the collection of mean log_10_ LC50s from the individual species-subsets, where in this case the subsets are defined according the aforementioned quasi-grouping. By making this assumption, we can envisage the quasi-taxonomic group means as a random sample from a hyper-population. In other words, on a log_10_ scale each quasi-taxonomic group SSD mean parameter is assumed to come from a normal distribution with a grand mean parameter (a measure of the overall sensitivity for the assemblage) and standard deviation – the hyper-parameters for the model.

Fitting a Bayesian model requires the specification of prior distributions over parameters. We used standard non-informative prior distributions: over the homogenous per-quasi-taxonomic SSD variance parameter (Inverse-Gamma with shape and rate parameter set to 0.001); over the hyper-population grand mean (a normal distribution with mean zero and variance 10^6^). The hyper-population standard deviation was assigned a Uniform (0, 10) distribution based on the reasoning of Gelman [36]. A basic sensitivity analysis to the prior distributions (Gamma vs. uniform etc.) was made based on quasi-taxonomic grouping, and no significant differences were found. Analysis was performed using Markov chain Monte Carlo sampling applied in R2WinBUGS [37] in R [38].

## Results

### Comparisons of the Species Tested from the Regions

**Figure 2:**
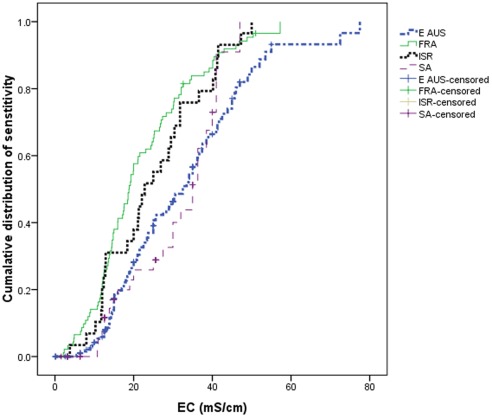
Kaplan-Meier estimated of cumulative distribution functions of 72 h LC_50_ values species (or species sensitivity distributions, SSDs) from each of the four regions (E AUS = eastern Australia, FRA = France, ISR = Israel and SA = South Africa).

In general, the macroinvertebrates tested in eastern Australia and South Africa were more tolerant than those tested from France and Israel (Figure 2, Table 1). There were statistically significant pair-wise differences in the Kaplan-Meier functions between the species tested in eastern Australia and France (χ^2^ = 26.532, P<0.001), eastern Australia and Israel (χ^2^ = 8.156, P = 0.004) but not eastern Australia and South Africa (χ^2^ = 0.053, P = 0.818). Likewise the Kaplan-Meier functions from France and Israel were not statistically different (χ^2^ = 0.359, P = 0.549). The South African Kaplan-Meier function was statistically different from those in France (χ^2^ = 9.495, P = 0.002) but not Israel (χ^2^ = 2.295, P = 0.130). The mean 72 h LC_50_ values of species tested in eastern Australia and South Africa were significantly higher than in the other regions (Table 1). Additionally the hazardous concentration (HC) for 1%, 5%, 10% and 20% of taxa having their LC_50_ exceeded was consistently higher for eastern Australia and South Africa than France and Israel (Table 1). For example, the French HC_5_ is 45% lower than its value for eastern Australia.

**Table 1:** Summary statistics of 72 h LC_50_ values (mS/cm) estimated from Kaplan-Meier functions for the different regions and various taxa.

	Range	Mean (95%CI)	Median (95%CI)	HC_1_	HC_5_	HC_10_	HC_20_	n –point & interval	n - right censored	n - total
E Australia	5.5−77.5	33.3^a^ (30.1−36.6)	32.5 (28.2−36.8)	6.2	10.4	13.8	16.4	106	100	206
France	2.2−57.3	22.3^b^ (19.5−25.0)	18.8 (16.6−21.0)	2.2	4.7	7.3	11.8	89	3	92
Israel	3.8−50.0	24.9^b^ (19.5−26.6)	22.8 (16.3−29.3)	<3.8	3.8−8	8.0−10.3	11.8−12	29	0	29
S Africa	10.8−47	31.4^a^ (27.5−35.2)	35.0 (30.7−39.2)	<10.8	10.8−11	11−12.6	16.0	24	26	50
Rare	2.2−77.5	33.6^a^ (30.6−36.5)	32.5 (29.3−35.7)	2.3	9.5−10.8	12.8−13.3	16.4	132	129	261
Abundant	3.8−52	22.3^b^ (29.2−24.4)	19.0 (17.7−22.1)	3.8	6.5	9.3−10	11.9	116	116	0
Crustaceans	11−77.5	39.6^a^ (32.9−49.3)	40.5 (30.1−51.0)	<11	11−13.5	14.8	21−24	25	6	31
Insects &Hydracarina	2.1−57.3	29.1^b^ (27.3−30.9)	28 (25.8−32.0)	3.3	8.4	12.6	14.6	187	114	301
Non-arthropods	4.6−37.9	15.6^b^ (13.2−18.0)	13.8 (11.7−15.9)	<4.7	6.5	9.0	10.8	36	9	45
Annelids	9.3−17.5	13.7^a^ (11.7−15.6)	13.8 (11.2−16.4)	<9.3	<9.3	<9.3	9.3−11.4	8	1	9
Insects	2.2−57.2	29.0^b^ (27.2−30.9)	28.9 (25.6−32.2)	3.3	8.4	12.5	15.0	177	107	284
Hydracarina	19.3−45.0	30.1^b^ (25.2−34.9)	29.1 (24.6−33.6)	<19.3	<19.3	19.3−20.9	20.9−21.5	10	7	17
Molluscs	4.6−37.9	17.2^a^ (13.6−20.7)	13.8 (10.2−17.4)	<4.6	4.6−6.5	9.0	10.8	22	8	30
Turbellaria	8−14.4	11.9^a^ (10.0−13.8)	11.6 (9.9−13.3)	<8.0	<8.0	<8.8	8.0−10.0	6	0	6
Coleoptera	13.6−53.5	38.0^a^ (34.2−41.7)	40.2 (36.0−44.4)	<13.6	17.6	18.8	23.4	29	36	65
Diptera	7.8−57.2	25.0^b^ (19.0−31.0)	21.1 (17.5−24.7)	<7.8	7.8−8.0	8.0−10.0	14.7	19	13	32
Ephemeroptera	2.4−>20	12.0^c^ (10.2−13.7)	12.8 (12.0−13.6)	<2.4	2.4−3.3	3.8−4.8	6.2−6.8	27	9	36
Hemiptera	10.8−>51	27.5^b^ (23.8−31.2)	25.3 (23.3−27.3)	<10.8	10.8−12.4	14.0−15.0	17.5−18.4	27	12	39
Lepidoptera	18.0−24.3*							1	0	1
Megaloptera	41.6							1	0	1
Odonata	12.6−55	38.0^a^ (35.3−40.7)	36.3 (30.0−42.6)	<12.6	21−27.5	29.5−30	30.3	32	24	56
Plecoptera	9.1−36.7	21.5^b^ (14.4−28.6)	18.3 (17.3−19.3)	<9.1	<9.1	<9.1	9.1−12.6	9	2	45
Trichoptera	2.2−32.0	19.6^b^ (17.5−21.8)	19.1 (17.2−21.1)	<2.2	8.4	11.8	14.4	34	11	45
Overall	2.2−77.5	28.9 (27.1−30.8)	27.0 (24.3−29.7)	3.0	8.2	11.2	13.9	248	129	377

HC_p_ is the estimated hazardous concentration (in terms of 72 h LC_50_ values) for p% of species tested and n is the number of samples. Mean values with the same letter are not statistically significant different from each other at the 0.05 level.

* Single species with an interval estimate of its LC50 value.

### Comparisons Between Groups of Taxa

The SSD for rare species (defined as those where there were always <50 individuals collected per collection episode) was to the right (more tolerant) of the SSD for abundant species (χ^2^ = 42.228, P<0.001) and the mean 72 h LC_50_ value of rare species was greater than that of common species (Table 1).

**Figure 3:**
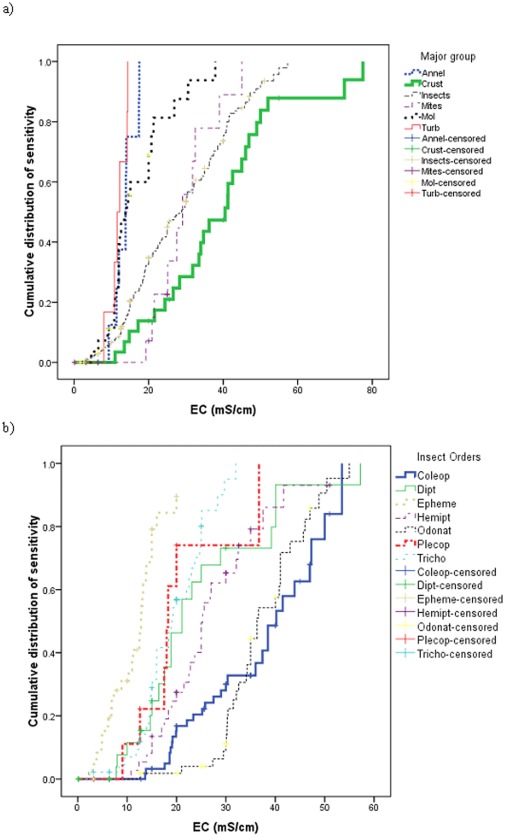
Cumulative distribution of 72 h LC_50_ values species of major taxomic groups.

Non-arthropods tended to be more salt sensitive than insects and Hydracarina (water mites), while crustaceans tended to be more tolerant still (Table 1). There were statistically significant differences in the Kaplan-Meier functions between these groups (χ^2^ = 7.732 to 69.162, P<0.001 to 0.005). Breaking these groups down further, there were similar Kaplan-Meier functions for annelids (segmented worms), molluscs and turbellaria (flatworms) (Figure 3A, Table 1) and these distributions mostly showed no statistically significant differences (χ^2^ = 0.749 to1.804, P = 0.179 to 0.387). The exception was that molluscs were more tolerant than turbellaria (χ^2^ = 3.878, P = 0.049). We do note that the number of annelid and turbellaria taxa examined was low (9 and 7, respectively) and that the SSDs, as represented by the Kaplan-Meier functions, for these taxa have to be considered as preliminary. Both Hydracarina and insects were more tolerant than all of the aforementioned non-arthropod groups (χ^2^ = 12.439 to 39.887, P<0.001) but there was no statistically significant difference in the Kaplan-Meier functions of Hydracarina and insects (χ^2^ = 0.010, P = 0.920). It is, however, noteworthy that there were no salt sensitive Hydracarina observed with the most sensitive mite’s 72 h LC_50_ value being 19.3 mS/cm (Table 1), in contrast 32% of insects have 72 h LC_50_ values ≤19.3 mS/cm (Figure 3A). Crustaceans tended to be more tolerant still than both insects and Hydracarina (χ^2^ = 4.561 to 7.334, P = 0.007 to 0.033).

Considering SSDs of insects only, Ephemeroptera was the most salt sensitive order (Figure 3B, Table 1) being statistically different from every other insect orders (χ^2^ = 4.176 to 77.266, P<0.001 to 0.041). Trichoptera and Plecoptera were the next most sensitive orders. The difference in the Kaplan-Meier functions of these two orders (χ^2^ = 0.069, P = 0.793) was not statistically significant. Trichoptera was statistically more sensitive than all remaining orders (χ^2^ = 12.854 to 49.948, P>0.001) except Diptera (χ^2^ = 2.462, P = 0.117). Plecoptera was more sensitive of all remaining orders (χ^2^ = 21.779 to 21.983, P<0.001) except Diptera (χ^2^ = 1.189, P = 0.275) and Hemiptera (χ^2^ = 3.592, P = 0.058). The Kaplan-Meier functions for Diptera and Hemiptera were similar (Figure 3B) and were not statistically significant different (χ^2^ = 0.685, P = 0.408). Both Diptera and Hemiptera were more sensitive than Coleoptera and Odonata (χ^2^ = 11.062 to 15.054, P≤0.001). Finally the Kaplan-Meier functions for Coleoptera and Odonata were not statistically significant from each other (χ^2^ = 0.161, P = 0.688). Only one species of each of Lepidoptera and Megaloptera (72 h LC_50_ values of 18.0–24.3 mS/cm and 41.6 mS/cm, respectively) was tested (Table 1) and while statistical comparisons are not made, the Lepidoptera species is moderately tolerant and the megalopteran fairly tolerant (Figure 3B).

Considering the mean LC_50_ value of particular taxa, showed the same trends observed as with Kaplan-Meier functions (Table 1).

### Comparison Between Regions Taking into Account Taxonomic Identity

Although there were differences in the Kaplan-Meier functions between regions (Figure 2), there were larger differences in the Kaplan-Meier functions for different taxonomic groups (Figure 3A, 3B). The number of species from each taxonomic group varied between the different regions. As we attempted to take representative sample of species for testing in each region, this variation should (at least partly) reflect the stream fauna present in each region. Therefore it is possible that the observed differences in the Kaplan-Meier functions between the regions are caused by the regions supporting differing richness of particular taxa [20].

Species were split into 7 groups: (1) Coleoptera & Odonata, (2) Crustaceans, (3) Diptera & Hemiptera, (4) Ephemeroptera, (5) Hydracarina, (6) non-arthropods, and (7) Trichoptera and Plecoptera. The groups with multiple taxa were chosen as there was no evidence that these taxa had different distributions of 72 h LC_50_ values (see above) and to maximise the sample size (species) in each group.

**Figure 4:**
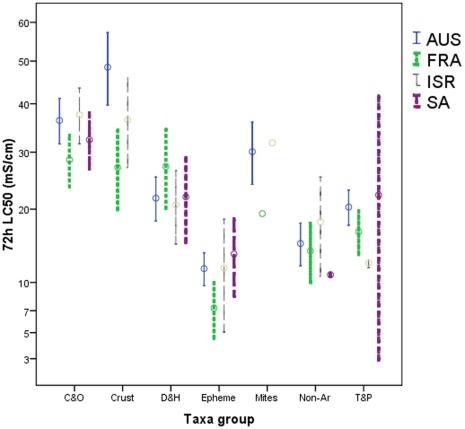
Mean ±2 standard errors (on both sides of the mean) of non-censored 72 h LC50 values for selected taxa groups in the four regions. Where C&O = Coleoptera and Odonata, Crust = Crustaceans, D&H = Diptera and Hemiptera, Epheme = phemeroptera, Mites = Hydracarina, Non-Ar = non-arthropods, T&P = Trichoptera and Plecoptera.

First considering only species with non-censored LC50 values, there was no statistical difference in the mean (square root transformed) 72 h LC_50_ values between the regions (F_3,191_ = 1.825, P = 0.144) but there were highly statistically significant differences between the taxa groups (F_4,191_ = 38.067, P<0.001). There was also no significant interaction between these factors (F_12,191_ = 1.517, P = 0.121). Hydaracarina and crustaceans were not included in the above ANOVA as ≤1 non-censored LC_50_ value was available from one or more region. Considering crustaceans only, there was a significant difference between the regions (F_2,22_ = 7.961, P = 0.003), with the mean (square root transformed) 72 h LC_50_ values of crustaceans being higher in eastern Australia than France but not Israel (Figure 4). The lack of replicated Hydracarina in France and Israel precludes statistical analysis, but the Israeli species tested had a similar salinity tolerance of the mean of those tested in eastern Australia, while the species tested in France were more sensitive than the mean of those tested in eastern Australia (Figure 4).

Considering now all species (regardless of their LC_50_ censored status) mean 72 h LC_50_ values calculated from the Kaplan-Meier functions for Coleoptera & Odonata, Crustaceans, Ephemeroptera, Non-Arthropods and Trichoptera & Plecoptera differed between some of the regions (Table 2). Additionally for these groups, except Non-Arthropods, there were statistically significant differences in the Kaplan-Meier functions between the regions (Table 2). For all taxa groups with differences in mean LC_50_ values between regions the mean 72 h LC_50_ value was highest in Eastern Australia, significantly lower in France (for Coleoptera & Odonata, Crustaceans and Ephemeroptera), Israel (for Trichoptera & Plecoptera) or South Africa (for Non-Arthropods). For other pair-wise comparisons between regions there were no significant differences, at the 0.05 level. The same order of relatively sensitivities of the taxa groups between regions, as shown for the mean LC_50_ values (Table 1, Figure 4), was repeated for the Kaplan-Meier functions.

**Table 2:** Mean 72 h LC_50_ values (mS/cm) estimated from Kaplan-Meier functions of the taxa groups in each of the regions.

	E Aust	France	Israel	S Africa	K-M functions (df = 3)#
C&O*	43.0^a^	29.7^b^	37.6^a,b^	35.8^a,b^	κ^2^ = 13.440, P = 0.004
Crust	51.1^a^	27.1^b^	36.5^a,b^		κ^2^ = 14.287, P = 0.003
D&H	25.1^a^	30.4^a^	20.7^a^	25.0^a^	κ^2^ = 4.153, P = 0.245
Epheme	14.1^a^	7.3^b^	11.8^a,b^	13.5^a,b^	κ^2^ = 13.089, P = 0.004
Mites	30.9	19.3	31.8		
Non-Ar	16.7^a^	13.9^a,b^	18.1^a,b^	10.9^b^	κ^2^ = 7.127, P = 0.068
T&P	22.4^a^	16.6^a^	12.3^b^	22.3^a,b^	κ^2^ = 24.898, P<0.001

Values with different superscript letters are significantly different (at the 0.05 level) between the regions.

# Statistical test of equality of the Kaplan-Meier functions between the regions.

* C&O = Coleoptera and Odonata, Crust = Crustaceans, D&H = Diptera and Hemiptera, Epheme = Ephemeroptera, Mites = Hydracarina, Non-Ar = non-arthropods, T&P = Trichoptera and Plecoptera.

There were too few species within particular families and genera tested across all regions to permit formal statistical analysis of differences in 72 h LC_50_ values at the family and genus levels. Considering the number of species tested and the standard deviation there is no evidence of any differences between families or genera. For example, a *Baetis* sp. in Eastern Australia had a 72 h LC_50_ value of 6.2 mS/cm in France the mean value of two species of this genus was 10.3 mS/cm. But given a standard deviation of 4.2 mS/cm in the 72 h LC_50_ value of the French *Baetis* spp. there is no evidence of a difference. Indeed several genera or families had remarkable similar mean 72 h LC_50_ value between the regions (Table 3). For example, Dugesiidae’s 72 h LC_50_ values was 11.1 mS/cm in eastern Australia and 11.9 in France. On the data available, there are no apparent large differences in the 72 h LC_50_ values of specific genera or families between the regions examined. Moreover, 72 h LC_50_ values for genera or families there were not necessarily higher in the more arid regions. *Baetis* was more salt sensitive in Eastern Australia and Israel than in France and *Micronecta* was more salt sensitive in Eastern Australia and South Africa than in France.

**Table 3:** Mean (and standard deviations) of 72 h LC_50_ values (mS/cm) of non-censored LC50 values within families and genera in each of the regions.

Taxa	E Aust	France	Israel	S Africa
Baetis	6.2, n = 1	10.3 (4.2) n = 2	7.1 (4.6), n = 2	
Hydropsyche		16.9 (2.5), n = 4	12.3 (0.71), n = 2	
Lestes			30.0 (0.63), n = 2	41, n = 1
Micronecta	17.1 (3.1), n = 4	27, n = 1		16.5 (3.5) n = 2
Physa acuta	15.1, n = 1	12.3, n = 1	12.0, n = 1	
Gammaridae		22.9 (11.1), n = 6	36.5 (6.7), n = 2	
Gomphidae	21.0, n = 1	30.6 (3.5), n = 3		23.8 (15.8), n = 2
Leptoceridae	21.9 (4.1), n = 9	19.6 (7.2), n = 2		32.0, n = 1
Notonectidae	27.8 (14.7), n = 3	32.1, n = 1	20.6 (3.1), n = 2	25, n = 1
Baetidae	10.6 (4.6), n = 5	10.3 (4.2), n = 2	11.4 (8.2), n = 3	11, n = 1
Caenagrionidae	43.8 (7.9), n = 6	45.9 (4.1), n = 2	36.6, n = 1	
Corixidae	19.5 (4.2), n = 10	26.2 (1.2), n = 2		16.5 (3.5), n = 2
Dugesiidae	11.1 (4.4), n = 2	11.9 (0.42), n = 2		
Dytiscidae	37.8 (9.9), n = 6	25.2, n = 1	45.1 (6.9), n = 2	42.7 (6.0), n = 2

n = number of species tested.

### Are the Sample of Species with LC_50_ Values Representative of their Regions

**Figure 5:**
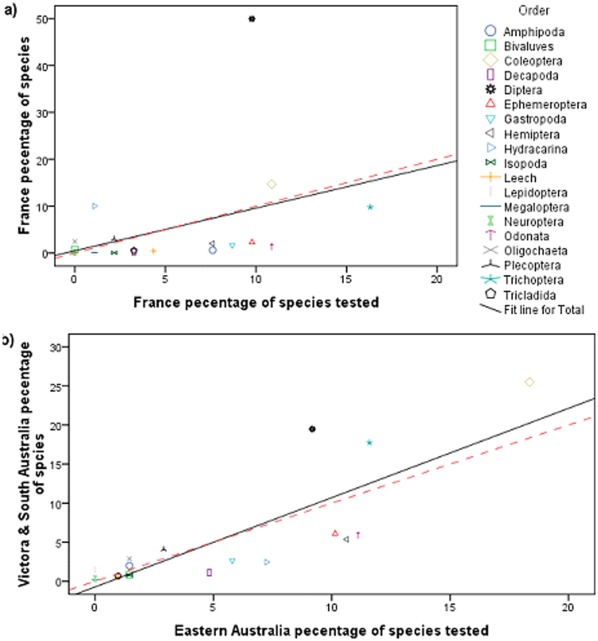
The relationship between the number of species in macroinvertebrate orders estimated for a region and the number of species tested (a) France and (b) eastern Australia. The solid straight black lines are the least squared regression lines and the dashed red lines are the equation y = x.

Overall the proportion of species in each order (n = 19) estimated to be present in eastern Australia and France were positively correlated with the proportion of species tested in these locations (r = 0.824, P<0.001 and r = 0.375, P = 0.113, respectively), although this correlation was not statistically significant in France. In both locations, the line of best fit was very similar to the one to one line (y = x) (Figure 5). So on average the number of species selected for testing from each order was in proportion to the richness of their order in the relevant region. However, especially in France, there were several orders which were under- or over tested relative to their richness. We estimate that 50% of the stream macroinvertebrates in France are dipterans, yet only 9.8% of the taxa tested in France were from that order (Figure 5A). Excluding Diptera, the correlation between the proportion of species in France and the proportion tested was statistically significance (r = 0.468, P = 0.0499). Hydracarina and Coleoptera were also somewhat under-tested in France, while Leaches, Amphipods, Hemiptera, Gastropods, Ephemeroptera, Odonata and Trichoptera were somewhat over-tested. In eastern Australia the misrepresentations were generally less than for France. Nevertheless, Diptera and Trichoptera were somewhat under-tested and Decapoda, Gastropods, Hydracarina, Ephemeroptera, Hemiptera and Odonata somewhat over-tested (Figure 5B).

### The Relative Richness of Orders Between the Regions

**Figure 6:**
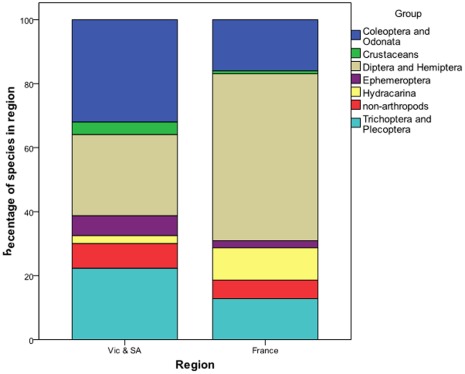
The Percentage of species in each of the quasi-taxonomic groups in Victoria and South Australia and France.

The quasi-taxonomic groups Diptera & Hemiptera and Hydracarina were relatively richer in France than in Eastern Australia (Figure 6). Non-arthropods at 6% and 8% of the species in France and Eastern Australia, respectively occupied a similar proportion of the taxa. The other groups (Coleoptera & Odonata, Crustaceans, Ephemeroptera and Trichoptera & Plecoptera) had relatively more species in Eastern Australia than France.

### Does the Acute Lethal Salinity Tolerance of Stream Macroinvertebrates Differ Between France and Eastern Australia?

**Figure 7:**
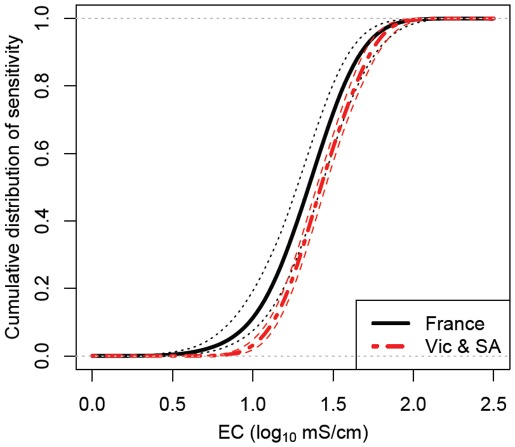
Distributions of species sensitivity in France and Victoria & South Australia from the Bayesian model. Heavy lines are the median estimate and the finer lines are 95% credibility intervals. Note the log_10_ scale of the x-axis.

The Bayesian model (Figure 1, Table 4) found the stream macroinvertebrate assemblage in France is more salinity sensitive to the than that in Victoria and South Australia (Figure 7). However, the shape of the two distributions is very similar (Figure 7). The difference is enough to result in practically important differences in protection concentrations in terms of 72-h LC50 values. The median estimate of HC_1_, for example, for France was approximately half that for Victoria and South Australia (Table 5). However, when it comes to the median (HC_50_) sensitivity both regions were more similar (Table 5, Figure 7).

**Table 4:** Summary of posterior distribution for parameters in the Bayesian model (see Figure 1) in France and Victoria & South Australia.

Region	Parameter	Posterior mean	Posterior S.D.	95% Credibility Interval#
France	Hyper-pop mean	1.26	0.105	(1.05, 1.47)
	Hyper-pop variance	0.0868	0.0835	(0.0211, 0.270)
	SSD variance	0.0517	0.00859	(0.0377, 0.0714)
Vic & SA	Hyper-pop mean	1.41	0.114	(1.18, 1.65)
	Hyper-pop variance	0.0893	0.096	(0.0201, 0.336)
	SSD variance	0.0230	0.00341	(0.0173, 0.0305)

Values are given to three significant figures in log_10_ mS/cm.

# Bayesian analysis produced credibility internal instead of confidence interval estimated by frequents analysis.

**Table 5:** Hazardous concentrations (HC), in terms of 72 h LC50 values (mS/cm), for various percentiles of species as estimated from the Bayesian model (see Figure 1) that estimate the distribution of 72 h LC50 values in France and Victoria & South Australia independent of the selection of species for testing.

	France	Vic & SA
HC_1_	4.07 (3.06, 5.09)	8.08 (7.09, 9.09)
HC_5_	7.21 (5.46, 8.51)	11.1 (10.1, 12.1)
HC_10_	9.51 (7.23, 11.3)	13.3 (12.3, 14.3)
HC_20_	12.9 (10.2, 15.5)	16.8 (15.7, 17.9)
HC_50_	22.2 (18.8, 26.7)	26.5 (24.8, 28.4)

Values given are median estimate (and 95% credible interval) to three significant figures.

## Discussion

Regardless as to how examined, we find some differences in the acute lethal salinity sensitivity (72 h-LC_50_ values) of stream macroinvertebrates between the regions. In terms of simply comparing all species tested between the regions the mean 72 h-LC_50_ value was approximately 10 mS/cm greater in Eastern Australia and South Africa than in France and Israel (Table 1). There were, however, greater differences in the 72 h-LC_50_ values between taxonomic groups (Table 1, Figure 3) with a 27 mS/cm difference in the mean 72 h-LC_50_ between Ephemeroptera and Crustaceans. Even still, the following quasi-taxonomic groups had higher mean 72 h-LC50 in Eastern Australia than France: Coleoptera & Odonata, Crustaceans and Ephemeroptera (Table 2), while the groupTrichoptera & Plecoptera was more tolerant in Eastern Australia and France than in Israel and non-Arthropods were more tolerant in Eastern Australia relative to South Africa. After weighting the quasi-taxonomic groups to take into account their relative species richness in the regions eastern Australia (Victoria and South Australia) and France (Figure 6), the resulting SSD for Victoria and South Australia was more salinity tolerant than that for France (Figure 7).

So suggestions [18], [19] that the aquatic biota of Australia is more salt tolerant relative to some other regions is supported by the current study. However, salinity tolerances of the invertebrates were similar in eastern Australia and the South Africa. Thus Australian stream macroinvertebrates are not more salt tolerant than those from all regions [20].

Furthermore, it is not as simple as the relative arid regions of Eastern Australia, South Africa and Israel supporting a more salt tolerant macroinvertebrate fauna than wetter France as previously suggested [20]. The group Trichoptera & Plecoptera, for example, was equally tolerant in both Eastern Australia and France but more sensitive in Israel. Furthermore Israel is located in an (semi-)arid region, yet for all species tested (Table 1) mean 72-h LC50 values of Israel species were similar to those in France and less than those in Eastern Australia. There was also no evidence that the sensitivity of the group Diptera & Hemiptera differed between any of the regions. Thus while in general eastern Australian macroinvertebrates may be more tolerant than those from some other regions e.g. France, it does not apply for all taxa and it does not appear to be simply related to aridity. Thus the greater salinity tolerance in eastern Australia may not be due to evolutionary factors and could (partly) reflect different ancestral communities between continents.

There are probably regions of the world where stream invertebrates do have different salinity tolerances to those regions examined in the current study. Although no experimental data on salinity tolerance are available for stream invertebrates of south-western Australia they have been recorded at salinity substantially higher, up to 192 mS/cm [39], than the 72-LC_50_ values reported here. Kay et al. [39] suggested that the high salinity tolerance in their region was the result of long period of salinization in south-western Australia (100 000’s of years) leading to the evolution of greater salinity tolerance. The current study, however, found no evidence of a generalised increased salinity tolerance across several taxa (Table 3) in any of the three regions where there is some history of salinization (eastern Australia, South Africa and Israel) relative to France where there is no history of salinization. Perhaps there has been a shorter history of salinization in eastern Australia, South Africa and Israel than south-western Australia. So an evolved salinity tolerance has not yet had a chance to occur. An alternative explanation is that in south-western Australia salt sensitive species have been eliminated leaving only salt tolerant species [19] and salt sensitive taxa have not colonised this region. This explanation, however, seems unlikely as it would imply there would be some species in other regions with salinity tolerances at ≈ 192 mS/cm which is 2.5 fold higher than the highest 72-h LC_50_ value found in the current study.

The existence of differences in 72-h LC_50_ values between the regions, especially within some of the quasi-taxonomic groups, shows that data on the sensitivity of local species are preferable to data from species from distant regions. However, we observed that variation in 72-h LC_50_ values was greater between taxonomic groups than between regions. It is thus more important that regional SSDs are reflective of the relative richness of taxonomic groups (i.e., Figure 6) than the species included in the SSD are obtained exclusively from the region. So we suggest in the absence of data from local species, that salinity sensitivity data from elsewhere will likely be suitable for construction of regional SSDs for salinity with two important provisos. Firstly, if there is a major difference in salinization history between regions, data across such regions are not interchangeable. It would not be appropriate, for example, to use the data from the 4 regions studied here in south-western Australia, or *vice verse*. Secondly, that the regional SSD is reflective of the relative richness of taxonomic groups in the region where inference from the SSD is to be made.

What approaches can ensure that regional SSDs are reflective of the relative richness of regional taxonomic groups? The use of rapid testing methods [15], [32] provided a representative sample of species in Victoria and South Australia. In France, however, rapid tests provided a poor sample of species, primarily due to ≈ 50% of species in France being dipterans but only ≈ 10% of species tested being from this order. Orders contained 0.06–50% of the total regional pool of species. Figure 5 illustrates the difficulties of obtaining a representative sample of species sensitivity. It is impossible to obtain a fully representative number of LC_50_ values for groups with few species, as even if only one species is tested, it is over-tested. While if no species from a group is tested, there is no experimental evidence of its sensitivity. We thus used a statistical model to adjust the importance of quasi-taxonomic groups to reflect their relative richness (Figure 6) and analyzed it using Bayesian methods in order to properly quantify uncertainty (Table 4). Thus the resultant SSDs (Figure 7) and hazardous concentration (Table 5) relate to the regional communities and not to the list of species that happened to be tested. We suggest that our, or similar [32], [35], [40], [41], models will be necessary to ensure that SSDs are reflective of species sensitivities of specific regions.

## Supporting Information

Table S1 **Salinity sensitivity data (µS/cm @ 25°C) collected from Israel.**
(PDF)

Table S2 **Salinity sensitivity data (mS/cm @ 25°C) collected for common species from France.**
(PDF)

Table S3 **Salinity sensitivity data (mS/cm @ 25°C) collected for rare species from France.**
(PDF)
