# Supplementary material for: Global Scale Variation in the Salinity Sensitivity of Riverine Macroinvertebrates: Eastern Australia, France, Israel and South Africa
Source: PLoS One. 2012 May 2;7(5):e35224. doi: 10.1371/journal.pone.0035224 (PMC3342278; doi:10.1371/journal.pone.0035224)
Supplement: Table S1 — Salinity sensitivity data (µS/cm @ 25°C) collected from Israel. (PDF) [file pone.0035224.s001.pdf]

Table S1. Salinity sensitivity data (µS/cm @ 25°C) collected from Israel.

Taxa	Order	Water body	Salinity at collection (µS/cm)	72h LC_{50} (µS/cm)	No. of Specimen × replicates
Laccophilus minutus	Coleoptera	Avi'el	1020	58200	10 × 3
Agabus biguttatus	Coleoptera	Tut	590	50000	12 × 3
Hydrochara dichromata	Coleoptera	Hadera	938	47300	13 × 1
Berosus dispar	Coleoptera	Ga'ash	402	42000	10 × 3
Haliptus lineatocollis	Coleoptera	Avi'el	1007	41500	12 × 3
Echinogammarus foxi	Amphipoda	Avi'el	1007	41250	15 × 3
Platycnemis sp.	Odonata	Avi'el	1020	40800	12 × 3
Pseudagrion sp.	Odonata	Avi'el	1020	36600	15 × 3
Hydracarina -adult	Acari	Ga'ash	350	35700	12 × 2
Gammarus syriacus	Amphipoda	HaShofet	679	31800	20 × 3
Lestes virens	Odonata	Hadera	874	30400	16 × 1
Lestes barbarus	Odonata	Hadera	874	29500	12 × 1
Culex pipiens	Diptera	Hadera	938	28900	15 × 3
Melanopsis buccinoidea	Archaeogastropoda	Zuqim	6500	28800	20 × 1
Hydracarina -young	Acari	Ga'ash	402	28000	13 × 2
Melanopsis buccinoidea	Archaeogastropoda	Hermon	433	25100	26 × 1
Culiseta longiareolata	Diptera	Tel-Aviv	1005	25000	15 × 3
Theodoxus michonii	Archaeogastropoda	Zuqim	6518	23000	20 × 1
Notonecta maculata	Hemiptera	HaShofet	671	22800	10 × 3
Laccophilus minutus -larva	Coleoptera	Avi'el	1034	22200	7 × 3
Chironomus sp.	Diptera	Hadera	874	21100	8 × 3
Cloeon dipterum	Ephemeroptera	Dora	470	20000	14 × 2
Theodoxus michonii	Archaeogastropoda	Hermon	334	19700	25 × 1
Anisops sp.	Hemiptera	Dora	470	18400	14 × 3
Ostracoda	Ostracoda	Ga'ash	413	13300	10 × 3
Caenis sp.	Ephemeroptera	Avi'el	1034	12900	16 × 3
Hydropsyche sp.	Trichoptera	Divsha	334	12800	10 × 2
Hydroptila sp.	Trichoptera	Avi'el	1034	12400	10 × 2
Cyzicus sp.	Branchiopoda	Hadera	938	12100	7 × 3
Physella acuta	Archaeogastropoda	Ga'ash	413	12000	12 × 3
Gyraulus ehrenbergi	Archaeogastropoda	Avi'el	1020	12000	7 × 3
Hydropsyche sp.	Trichoptera	Avi'el	1034	11800	7 × 3
Baetis sp.	Ephemeroptera	Avi'el	1036	10300	13 × 2
Chirocephalus neumanni	Branchiopoda	Hadera	938	9500	20 × 1
Ostracoda	Ostracoda	Ga'ash	350	8800	12 × 3
Daphnia magna	Branchiopoda	Ga'ash	350	8400	20 × 3
Daphnia curvirostris	Branchiopoda	Hadera	874	8250	25 × 3
Simulium sp.	Diptera	HaShofet	679	8000	10 × 1
Daphnia magna	Branchiopoda	Ga'ash	413	7000	20 × 3
Baetis sp.	Ephemeroptera	Divsha	334	3800	10 × 2
Berosus dispar -larva	Coleoptera	Ga'ash	420	2200	10 × 1
Berosus dispar -eggs	Coleoptera	Ga'ash	420	1800	10 × 1
