# Supplementary material for: Global Scale Variation in the Salinity Sensitivity of Riverine Macroinvertebrates: Eastern Australia, France, Israel and South Africa
Source: PLoS One. 2012 May 2;7(5):e35224. doi: 10.1371/journal.pone.0035224 (PMC3342278; doi:10.1371/journal.pone.0035224)
Supplement: Table S2 — Salinity sensitivity data (mS/cm @ 25°C) collected for common species from France. (PDF) [file pone.0035224.s002.pdf]

Table S2. Salinity sensitivity data (mS/cm @ 25°C) collected for common species from France.

Comon		Family	Genus	Species	n	LC_{50} (mS/cm)
**Turbellaria**	**Tricladida**	Dugesidae	*Dugesia*	*D. polychroa*	180	11.6
				D. tigrina	144	12.2
**Gastropoda**	**Gastropod**	Hydrobiidae	*Potamopyrgus*	*P. antipodarum*	225	37.9
	**Prosobranchia**	Viviparidae	*Viviparus*	*V. contectus*	180	6.5
		Bithyniidae	*Bithynia*	*B. tentaculata*	168	11.8
		Neritidae	*Theodoxus*	*T. fluviatilis*	225	20
	**Pulmonate**	Ancylidae	*Ancylus*	*A. fluviatilis*	120	4.6
		Limnaeidae	*Radix*	*R. ovata*	210	12
**Hydracarina**				Hydracarina spp.	120	19.3
**Crustacea**	**Isopoda**	Asellidae	*Asellus*	*A. aquaticus*	210	21.6
			Proasellus	*P. meridianus*	215	24.4
	**Amphipoda**	Corophiidae	*Chelicorophium*	*C. curvispinum*	195	14.8
		Gammaridae	*Dikerogammarus*	*D. villosus*	195	26.6
			Echinogammarus	*E. berilloni*	139	28.3
			Gammarus	*G. roeseli*	210	11
				G. pulex	180	17.5
				G. tigrinus	165	40.5
				G. pulex	160	16.9
				G. fossarum	120	13.5
	**Decapoda**	Atyidae	*Athyaephyra*	*A. desmarestii*	180	34.7
**Insecta**	**Coleoptera**	Elmidae	*Esolus*	*E. parallelipipedus*	195	19.9
			Elmis sp.(A)	*Elmis* sp.(A)	100	20.2
			Elmis sp.(L)	*Elmis* sp.(L)	111	17.5
			Esolus	*E. angustatus* (A)	153	18.6
	**Diptera**	Chironomidae	*Chironomus*	*C. plumosus*	180	23.1
		Simulidae	*Simulini*	*Simulini* sp.	180	7.8
	**Ephemeroptera**	Baetidae	*Baetis*	*B. rhodani*	180	7.3
		Ephemerellidae	*Ephemerella*	*E. ignita*	195	4.9
		Potamanthidae	*Potamanthus*	*P. luteus*	225	13.8
	**Hemiptera**	Aphelocheiridae	*Aphelocheirus*	*A. aestivalis*	140	26.8
	**Odonata**	Caenagrionidae	*Caenagrion*	*C. puella*	150	43
		Placticnemididae	*Platycnemis*	*P. pennipes*	168	50.4
	**Trichoptera**	Hydropsychidae	*Hydropsyche*	*H. pellucidula*	195	19.3
				H. contubernalis	180	14.5
				H. exocellata	180	18.9
				H. siltalai	180	15
		Leptoceridae	*Athripsodes*	*A. cinereus*	150	24.7
