# Supplementary material for: Global Scale Variation in the Salinity Sensitivity of Riverine Macroinvertebrates: Eastern Australia, France, Israel and South Africa
Source: PLoS One. 2012 May 2;7(5):e35224. doi: 10.1371/journal.pone.0035224 (PMC3342278; doi:10.1371/journal.pone.0035224)
Supplement: Table S3 — Salinity sensitivity data (mS/cm @ 25°C) collected for rare species from France. (PDF) [file pone.0035224.s003.pdf]

Table S3. Salinity sensitivity data (mS/cm @ 25°C) collected for rare species from France.

Rare		Family	Genus	Species	n	LC_{50}
**Turbellaria**	**Tricladida**	Dendrocoelidae	*Dendrocoelum*	*D. lacteum*	21	12.8 - 16.0
**Annelida**	**Leech**	Erpobdellidae	*Erpobdella*	*E. testacea*	40	11.3 - 12.8
		Erpobdellidae	*Erpobdella*	*E. octoculata*	30	12.9 - 15.1
		Glossiphonidae	*Glossiphonia*	*G. complanata*	13	10.3 - 12.5
		Piscicolidae	*Piscicola*	*P. geometra*	25	15.8 - 19.0
**Crustacea**	**Decapoda**	Cambaridae	*Orconectes*	*O. limosus*	18	44.0 - 49.8
		Astacidae	*Astacus*	*A. astacus*	45	44.0 - 48.0
**Gastropoda**	**Pulmonate**	Lymnaeidae	*Lymnaea*	*L. stagnalis*	50	10.3 - 11.3
		Physidae	*Physa*	*P. acuta*	5	8.0 - 14.6
**Insecta**	**Coleoptera**	Gyrinidae	*Orectochilus*	*O. villosus* (L)	10	27.4 - 33.3
		Elmidae	*Esolus*	*E. angustatus* (L)	14	18.6
		Elmidae	*Stenelmis*	*S. canaliculata*	3	14.8 - 20.4
		Dysticidae	*Platambus*	*P. maculatus* (L)	2	20.6 - 29.8
		Dysticidae	*Dysticus*	*D. marginalis* (A)	2	>51.0
		Scirtidae	*Elodes*	*Elodes* sp. (L)	14	19.2
	**Diptera**	Athericidae	*Atheryx*	*Atheryx* sp.	6	35.1 - 45.1
			Athricops	*A. crassipes*	10	35.1 - 45.1
		Empididae		Empididae spp.	12	39.2
		Limoniidae	*Pediciini*	*P. dicranota*	45	10.8 - 14.3
			Hexatomini	*Hexatoma* sp.	3	19.2 - 23.0
		Tabanidae		Tabanidae spp.	10	54.7 - 59.8
		Tipulidae		Tipulidae spp.	13	15.1 - 20.0
	**Ephemeroptera**	Baetidae	*Baetis*	*B. niger*	12	12.0 - 14.5
		Ephemeridae	*Ephemera*	*E. danica*	4	2.5 - 4.1
				E. vulgata	5	6.8
		Heptageneidae	*Rhithrogena*	*R. semicolorata*	3	2.8 - 6.8
		Oligoneuriellidae	*Oligoneuriella*	*O. rhenana*	21	1.8 - 2.9
	**Hemiptera**	Corixidae	*Micronecta*	*M. poweri*	5	25.0 - 29.0
			Sigara	*Sigara* sp.	2	20.6 - 30.0
		Heptageneidae	*Naucoris*	*N. maculatus*	1	>32.6
		Nepidae	*Nepa*	*N. cinerea*	2	8.0 - 16.9
			Ranatra	*R. linearis*	6	>51.0
		Notonectidae	*Notonecta*	*N. glauca*	6	29.7 - 34.5
	**Lepidoptera**	Crambidae	*Acentria*	*A. ephemerella*	4	18.0 - 24.3
	**Megaloptera**	Sialidae	*Sialis*	*S. lutaria*	21	39.2 - 44.0
	**Odonata**	Aeshnidae	*Boyera*	*B. irene*	2	29.7 - 34.5
		Caenagrionidae	*Ischnura*	*I. elegans*	9	47.8 - 49.9
		Calopterygidae	*Calopteryx*	*C. splendens*	55	27.5 - 32.7
				C. virgo	24	29.7 - 33.3
		Corduliidae	*Somatochlora*	*S. metallica*	8	32.6
		Gomphidae	*Onychogomphus*	*O. forcipatus*	5	32.8 - 35.8
			Gomphus	*G. pulchellus*	4	24.8 - 29.9
				G. vulgatissimus	3	27.5 - 32.9
	**Plecoptera**	Leuctridae	*Euleuctra*	*E. geniculata*	6	16.6 - 23.4
			Leuctra	*L. fusca*	27	7.3 - 10.8
	**Trichoptera**	Brachycentridae	*Brachycentrus*	*B. subnubilius*	3	26.6 - 32.8
		Lepidostomatidae	*Lepidostoma*	*L. hirtum*	8	14.8
		Leptoceridae	*Oecetis*	*O. ochracea*	6	10.4 - 18.7
		Limnephilidae	*Halesus*	*H. tessellatus*	16	12.9 - 19.0
			Micropterna	*M. lateralis*	10	7.5 - 9.3
			Potamophylax	*P. luctuosa*	3	12.9 - 19.0
		Polycentropodidae	*Neureclipsis*	*N. bimaculata*	21	16.9 - 20.0
			Cyrnus	*C. trimaculatus*	8	25.0
		Rhyacophilidae	*Rhyacophila*	*R. dorsalis*	6	1.8 - 2.5
		Sericostomatidae	*Sericostoma*	*S. personatum*	14	16.0
